# Collaborative Quality Improvement Strategy in Secondary Prevention of Cardiovascular Disease in India: Findings from a Multi-Stakeholder, Qualitative Study using Consolidated Framework for Implementation Research (CFIR)

**DOI:** 10.5334/gh.1161

**Published:** 2022-10-11

**Authors:** Kavita Singh, Mark D. Huffman, Leslie C. M. Johnson, Nikhil Tandon, Dorairaj Prabhakaran, Emily Mendenhall

**Affiliations:** 1 Public Health Foundation of India, Centre for Chronic Conditions, and Injuries, Gurugram, India; 2 Centre for Chronic Disease Control, Clinical Trials Unit, New Delhi, India; 3 Heidelberg Institute of Global Health, University of Heidelberg, Germany; 4 Washington University School of Medicine, St. Louis, USA; 5 Northwestern University Feinberg School of Medicine, Department of Medicine, Chicago, USA; 6 The George Institute for Global Health, University of New South Wales, Sydney, Australia; 7 Emory University School of Medicine, Department of Family and Preventive Medicine, Atlanta, USA; 8 All India Institute of Medical Sciences, Department of Endocrinology and Metabolism, New Delhi, India; 9 Edmund A. Walsh School of Foreign Service, Georgetown University, Washington, USA

**Keywords:** Cardiovascular disease, collaborative care, secondary prevention, India

## Abstract

**Background:**

Cardiovascular disease (CVD) is highly prevalent in India, and little is known about the perception of patients and providers about a package of collaborative quality improvement (C-QIP) strategies consisting of provider-focused electronic health records-decision support system (EHR-DSS), non-physician health workers (NPHW), and patient-facing text messages to enhance the CVD care.

**Objective:**

To explore the barriers and enablers of the C-QIP strategy from the perspective of providers, health administrators, patients, and care givers in India.

**Methods:**

We conducted a qualitative study using the consolidated framework for implementation research (CFIR) to understand the challenges and facilitators of implementing C-QIP strategy to enhance CVD care in the Indian context. A diverse sample of 38 physicians, 14 non-physician health workers (nurses, pharmacists), 4 health administrators, and 16 patients and their caregivers participated in semi-structured interviews. All interviews were audio-recorded, transcribed, translated, anonymised, and coded using MAXQDA software. We used the framework method and CFIR domains to analyze the qualitative data.

**Results:**

Barriers perceived from providers’ and health administrators’ perspectives in providing quality CVD care were high patient volume, physician burnout, lack of robust communication or referral system, paucity of electronic health records, lack of patient counsellors, polypharmacy, poor patient adherence to medications, and lack of financial incentives. Low health literacy, high cost of treatment, misinformation bias, and difficulty in maintaining lifestyle changes were barriers from patients’ perspectives. The CFIR identified key enablers for the implementation of C-QIP such as standardized treatment protocol, reduced medication errors, improved physician-patient relationships, and enhanced patient self-care through trained and supported NPHW. Barriers included: heterogenous healthcare settings, diverse patient groups and comorbidities, associated costs of care and interoperability, confidentiality, and data privacy issues around the use of EHR-DSS.

**Conclusion:**

Strategies to enhance CVD care must be low-cost, culturally acceptable, and integrated into existing care pathways.

## Introduction

Cardiovascular disease (CVD) is highly prevalent in India, with more than 70 million people affected in India, and 523 million globally [1,2,3]. CVD poses challenges for patients in both home and clinical settings to prevent disease-related morbidity and mortality. People with CVD must navigate modified lifestyle habits and a different way of seeking medical care. How people face these self-care challenges are varied, and important gender, socio-economic class and caste differences exist, which manifest in how people manage CVD. These differences also exist in terms of how people access and adhere to prescribed therapy, which further exacerbates inequities in health outcomes [4,5,6]. Tackling quality of health care is as critical as addressing barriers to access care for people with CVD. A 2018 systematic analysis of deaths in 137 countries found that poor quality of health care led to a larger burden of mortality than low access to care [7]. Furthermore, the National Academy of Medicine 2018 report on Global Quality Chasm underscored the urgency for comprehensive efforts to close health care quality gaps globally [8].

Quality improvement (QI) strategies involving clinical decision support, audit and feedback reports, and team-based care have been successful in well-resourced settings for improving care [9]. However, little is known about trust, acceptability, and feasibility of such QI strategies for CVD care among patients and providers in India. Team-based care involving specialists and community health workers to empower, encourage, and facilitate care processes for patients with acute coronary syndrome (ACS) has shown improvements in medication adherence and systolic blood pressure [10]. Prior studies using these QI strategies have also shown improvements in cardiovascular risk factor control, prescription of evidence-based medications, and quality of life among patients with ACS or stroke and among those at high CVD risk [11,12,13]. The current study builds on this existing evidence of success and leverages pilot work on task-sharing and technology interventions to further inform the development of the multifaceted **c**ollaborative **q**uality **i**m**p**rovement (C-QIP) strategy (combining task-sharing and technology) for chronic care of CVD in India.

In the Indian context, several factors influence the CVD care gaps such as economic burden on patient and caregivers, low health literacy, and lack of health system infrastructure [14,15]. To improve uptake of proven strategies for CVD care, skilled non-physicians’ health workers (NPHW) and technology (such as electronic health records-decision support system [EHR-DSS], text-messages) offer an achievable and potentially low-cost care delivery model. Involvement of trained and supervised NPHW can support patient self-management, and follow-up, navigation of clinical services, and treatment coordination. EHR-DSS offers a centralized patient record system (stores patient medical history, lab tests, prescriptions, and self-care details) and may enhance timely treatment modification by the physician’s considering patient’s self-care habits (adherence to prescribed therapy, tobacco and alcohol use, diet, exercise, and psychosocial factors). Text-messages on healthy lifestyles can further improve self-care behaviors and serve as reminders for the next clinic visit and laboratory test to maintain continuity of care. Ethnographic and qualitative research have demonstrated how quality worsens over time in care for people with chronic conditions [16,17]. This study aimed to describe the challenges and opportunities around chronic care for CVD from the perspectives of providers and patients. Further, using consolidated framework for implementation research (CFIR) [18], we explored the perceived barriers and enablers to the implementation of a C-QIP strategy in India.

## Methods

### Design

This qualitative study was designed to inform development and implementation of the C-QIP strategy (consisting of EHR-DSS, NPHW, and text messages) to ensure that it addressed the myriad factors that can facilitate, as well as impede, collaborative care programs in India for patients with CVD. A prospective feasibility randomized controlled trial (RCT) will assess the effectiveness and acceptability of C-QIP strategy involving 400 patients with CVD attending four diverse public and private hospitals in India [19]. Since our goal was to standardize outpatient care delivery models for patients with CVD, we drew insights through semi-structured interviews with care providers, health administrators, patients, and caregivers in India. We drew upon key informants’ experiences from several clinics from different parts of India and focused on what worked and what did not within and around the QI strategies for CVD care. Further, we used qualitative interviews to capture how people perceived CVD management using QI strategy (such as use of EHR-DSS, NPHW, and patient-facing text messages to encourage healthy lifestyles), in their everyday lives from various perspectives, including at the provider, health administrators, patient, and caregiver levels. The data collected can inform implementation of the C-QIP strategy to address clinical quality, including related to patient-reported experiential measures – like feeling included or heard, being included and respected, and being recognized as a central or peripheral feature of the collaborative care – which may facilitate or impede success. The study was approved by the Institutional Ethics Committee of the Public Health Foundation of India. Before conducting the interview, informed consent was obtained from the participants, and interview data were deidentified to ensure confidentiality.

### Setting and participants

**Figure 1:**
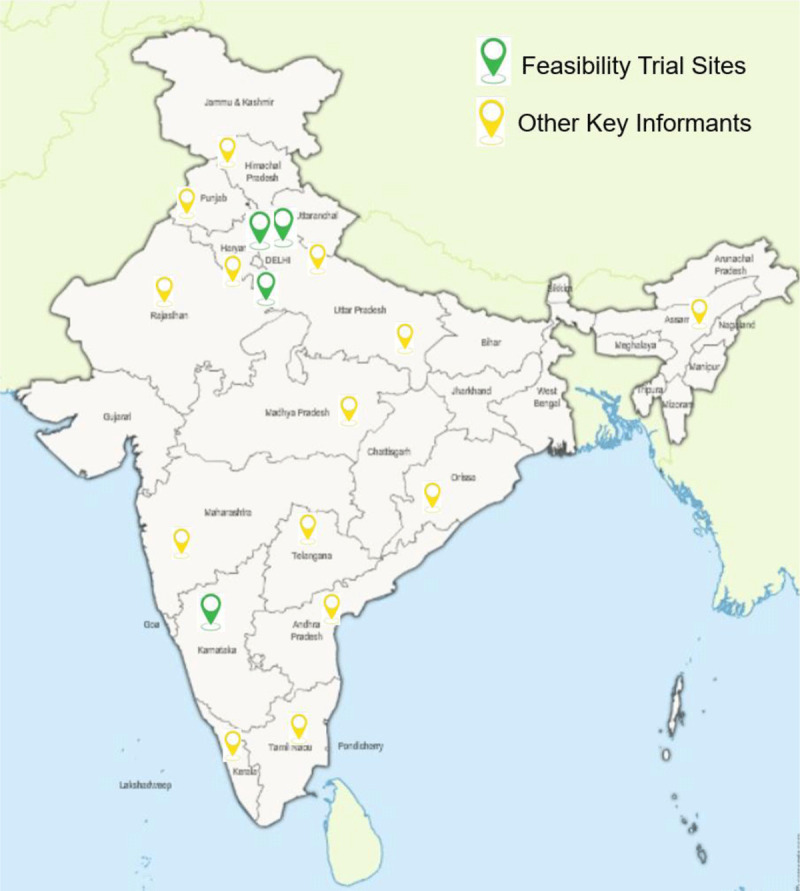
Location of participating sites and other key informants interviewed in the C-QIP study. * Patients, caregivers, and health administrators all were interviewed from the 4 feasibility trial sites. ** Physicians, cardiologists, and non-physician health workers (nurse, community health workers and pharmacists) were interviewed from both feasibility trial sites and other diverse clinical settings in India.

This study was conducted among three different groups of stakeholders: 1) health care providers, 2) health administrators, 3) patients, and their caregivers. Health care providers constituted physicians, cardiologists, nurses, community health workers, and pharmacists engaged in providing care for patients with CVD. We used a purposive sampling frame to select an initial sample of participants who had diverse roles and experiences in the care of patients with CVD ranging from cardiologists and physicians to cardiac care unit nurses, pharmacists, community health workers to health administrators and policy makers. We then used a snowballing sampling technique based on the in-depth interviews to recruit additional participants with increasing variability until we achieved theoretical saturation, the point at which no novel concepts seemed to emerge. The four hospitals selected for the C-QIP trial are large [19], tertiary care teaching hospitals with a mix of two government (All India Institute of Medical Sciences, and GB Pant Hospital, New Delhi, India) and two private hospitals (Sir Gangaram Hospital, New Delhi and SDM Hospital, Karnataka, India). The health care administrators, patients, and caregivers were interviewed from these four hospitals in Delhi and Karnataka. Figure 1 depicts the location of four hospital sites for the Feasibility RCT, and other key informants interviewed in this study.

## Data collection

We conducted semi-structured interviews in person and over the phone between September 2019 and February 2020 using an interview guide specifically developed for each participant group: healthcare provider, health administrator, patient, and caregiver (see supplement for the interview topic guide). The interview guide broadly focused on three different aspects of collaborative CVD care using CFIR constructs to elicit information on barriers and enablers of the C-QIP strategy in the Indian care setting. First, interview questions were asked about what challenges are faced by providers, administrators, patients, and their caregivers to manage CVD in India. Second, the interview questions delved into understanding how common CVDs are across age, gender, and socio-economic groups, as well as co-morbidities, patients’ needs and understanding of CVD and related self-care, and how providers motivate patients’ self-care. Third, interview questions focused on eliciting views of multiple health system stakeholders on the C-QIP strategy, perceived enablers, and barriers to its implementation in diverse clinical practices (public, and private), and broader relevance of QI strategies for CVD care in Indian context. Interview questions also probed the relative advantage, self-efficacy, and systems-level challenges or the cultural issues that might affect the implementation of the C-QIP strategy in India.

Three members of the study team (KS, VSB, and RD) conducted the in-depth interviews. In-depth interviews used open ended questions, and probes were used to elucidate emerging themes. All interviews with healthcare providers and health administrators were conducted in English, whereas patient and caregivers’ interviews were conducted in dyads and in the local languages (Hindi and Kannada) by a team member fluent in these languages. Interviews ranged from 30 minutes to more than one hour in length. Interviews were audiotaped, transcribed verbatim, and checked for their accuracy by study team members.

## Analysis

An iterative approach was used for data analysis based on the framework method for qualitative research [20]. The first author developed a codebook based on the interview guide, which was revised iteratively to include inductive codes. The transcripts were coded by two authors (KS, and VSB) to incorporate inductive codes and the initial codebook. To reach consensus on where discrepancies in coding the theme came up, we consulted senior authors (EM, DP, and MH). Once coding was complete, we then systematically evaluated the codes to see among whom and how they emerged; we used this analysis to develop a theoretical framework to describe key themes around barriers and enablers for implementing the C-QIP strategy in India. Further, interview data were analyzed from the lens of Consolidated Framework for Implementation Research (CFIR) to guide effective implementation of the C-QIP strategy [21,22]. Identified barriers and enablers were categorized according to the five CFIR domains and to organize CFIR constructs: intervention characteristics, characteristics of individuals, inner setting, outer setting, and process of implementation [23]. Intervention characteristics assessed key intervention attributes (e.g., relative advantage, adaptability, complexity, cost) known to influence implementation success. Inner setting assessed structural characteristics, networks and communication, readiness for implementation, whereas outer setting assessed the external policies that are known to influence implementation of new intervention. Characteristics of individuals involved assessed the knowledge, and belief of the implementation actors about the intervention. Lastly, process domain of CFIR assessed the engagement of stakeholders, and planning and evaluation of the C-QIP strategy to inform implementation. We used MAXQDA software for analysis and adhered to Consolidated criteria for Reporting Qualitative Research standards [24].

## Results

Among the three stakeholder groups (N = 72 participants), we interviewed 38 physicians (mean age: 56 ± 7.8 years, 88% men), 14 non-physician health workers (38 ± 4.2 years, 38% men), 4 health administrators or policy maker, 100% men with mean age: 54.4 ± 5.2 years, and 16 patients (52.4 ± 6.4 years, and 62% men) and their caregivers (32.4 ± 9.5 years, and 52% men). Two physicians and a patient refused to participate in the interview due to personal reasons. Physician- and hospital-level characteristics are summarized in Table 1. On average, physicians had >10 years of experience practicing cardiology, provided care for 550 patients per month, spending 15 minutes with patients at their initial visit, and 5–10 minutes in follow-up visits.

**Table 1:** Key informant’s (physicians) and hospital-level characteristics.


PHYSICIAN’S CHARACTERISTICS	TOTAL (N = 38)

Age (in years, mean ± SD)	55.9 ± 7.8

Men, n (%)	32 (87.5)

**Highest academic qualification**	

DM – Cardiology, n (%)	30 (78.9)

**Number of years practicing cardiology**	

5 – 10 years, n (%)	3 (0.8)

10 – 20 years, n (%)	11 (30.0)

>20 years, n (%)	25 (65.7)

**CVD management guidelines followed**	

ACC/AHA and ESC, n (%)	25 (65.7)

ACC/AHA, and ESC with Indian guidelines, n (%)	14 (36.8)

Others (subjective to patient case), n (%)	3 (0.8)

**Patient consultations per month**	

Median (IQR)	550 (420–700)

**Time spent at initial visit (in minutes)**	

Median (IQR)	15 (10–20)

**Time spent at follow up visit (in minutes)**	

Median (IQR)	10 (5–16)

**Consultation fee, Indian Rupees**	

Median (IQR)	325 (250–700)

**HEALTH FACILITY LEVEL FACTORS**	**TOTAL (N = 32)**

**Type of clinical setting**	

Individual Practice, n (%)	2 (6.25)

Group practice – Private, n (%)	3 (9.3)

Hospital – Government, n (%)	9 (28.1)

Hospital charity, n (%)	8 (21.8)

Hospital – Private for profit, n (%)	10 (31.2)

**Reminders for clinic appointments, yes**, n (%)	11 (34.3)

**Types of reminders sent to patients**	

Text message, n (%)	10 (31.2)

Phone, n (%)	1 (3.1)

**Availability of patient record maintenance facility, yes**, n (%)	27 (84.3)

**Patient electronic database system, yes**, n (%)	16 (50)

Availability of physician performance feedback system, yes, n (%)	15 (46.8)

**Use of CVD management strategies**	

Patient education materials (booklets, poster), n (%)	4 (12.5)

Individual CVD counselling, n (%)	10 (31.2)

Group education + individual counselling, n (%)	18 (56.2)


ACC = American College of Cardiology, AHA = American Heart Association, ESC = European Society of Cardiology, IQR = Inter quartile range, DM = Doctorate in Medicine, Cardiology, SD = standard deviation, IQR = interquartile range, CVD = cardiovascular disease.

### Current practices and challenges to CVD care in India

Table 2 summarizes salient barriers to chronic care of CVD from the provider’s and patient’s perspectives. Most physicians expressed *high patient volume, time-constraints, and low health education* among patients to be greatest challenges for providing quality CVD care. Physicians found insufficient time to provide care in the first place. One cardiologist explained, ‘Volume is too high that we are not able to spend time with each patient in a proper way.’ Further, there are *too few specialists* and too many patients that need specialty care. One out of every three providers identified ‘physician burnout’ to reflect, what they called, a ‘cultural syndrome’, described by one provider as when clinicians ‘are no longer interested to be actively involved in patient management’ because ‘the load is so much, every person has overloaded system, so in an overloaded system a single doctor cannot treat so many cases.’ He went on to explain, ‘most of the time is going in treating the patient and not in healing. Healing requires both preventive as well as therapeutic (efforts).’ This idea of remedial approaches was common, as many recognized that there is a lack of focus on prevention efforts such as tobacco cessation. This was recognized as an attitude problem of physicians: ‘The attitude needs to be changed. We wait for the disease to develop and all the efforts of all the corporate hospitals and everybody is just for the disease to occur so that now they can be rectified. There is nobody interested in preventing the disease.’ Lack of robust communication and referral systems, including paucity of electronic health records, emerged as barriers to quality CVD care as described by a health administrator: ‘I think there is lot of misguidances to the patient, as to how, when and where they should approach which specialty.’ Another physician added ‘There is no mechanism where we monitor the follow-up of the patient’, emphasizing a gap in care coordination.

**Table 2:** Most salient barriers to cardiovascular disease care in Indian context.


CATEGORY	SPECIFIC BARRIER	ILLUSTRATIVE QUOTES

**patient**	Low health literacy	‘I don’t know about (CVD) symptoms. Earlier I had an accident (when asked about the cause of CVD).’ (Patient-02)

	Socio-economic status (Poverty)	‘If they don’t have money to eat well the question about avoiding disease, (CVD) you know…so there is where the problem starts…mainly in lower economic status.’ (caregiver-02)

	Cost of care (affordability)	‘There is one medicine Vymada (medicine to treat heart failure), that one strip is around 1075 Rs. He has to take 60 tablets in a month. So, it’s around 4000 Rs for us. We sell milk, we sell crop then only we can get it. We are farmers.’ (Caregiver-04)

	Long queues	‘Here the line (for patient registration) starts midnight 2 am. So, I stand in line. Then they said they will make a card in morning 11 am. Cardiologist doctor comes here at 2pm. So, we got the number and showed to doctor.’ (Patient-04)

	Difficulties in maintaining self-care habits: misconceptions around diet, exercise, tobacco, alcohol use	‘The biggest misconception they have is they need to exercise only in the morning. And no benefit after that.’ (Physician-07)

	Competing obligations and lack of family support	‘With the fragmentation of the family, the family is getting nuclear, there are not many people to take care of elderly.’ (Caregiver 12)

**provider**	Time constraints and high patient volume	‘Sometimes we feel that one patient needs more time than the other, but we are always in the rush, finishing the rounds, coming here for the OPD, doing some ECHO, then the CATH lab, then again rounds, so I feel it is the shortage of time (Greatest barrier to chronic care of CVD). (Cardiologist-26)

	Mis-information epidemic	‘Many of the highly literate people who are computer savvy, they are (What I call them is misinformed) of this misinformation because of this misinformation going on in various social media so that misinformation epidemic must be controlled so sometimes that takes lots of time.’ (Physician-09)

	Lack of focus on prevention	‘We know what is killing us, we are not prepared to stop it because the smoking industry is more powerful than the few doctors who are concerned about it.’ (Cardiologist-04)

	Polypharmacy and poor adherence to medications	‘When you have 20 drugs, they must make sure that he understands which drugs are essential. So, it is a challenge.’ (Physician-09)

	Mixed recommendations from other health practitioners	“They (patients) have to follow one (doctor) and then follow the other one and sometimes there may be overlap of therapies which may not be properly addressed. (doctor).’ (Physician-12)

	Inadequate uptake of evidence-based guidelines	‘Lack of repeated upgradation of knowledge among the physicians. Many of the physicians are not keeping themselves updated with the knowledge. That is a very big problem.’ (Physician-09)

	Beliefs about traditional medicine practices	‘Some patients are having their own ideas about getting treatment from some alternative sources of medicine, so they don’t listen to us, and they take what they want.’ (Physician-14)

**health system**	Shortage of trained manpower	‘I think number one challenge is the (limited) availability of the (trained) manpower, so they need to have qualified people available in the hospital around the clock.’ (Health administrator-01) ‘We don’t have a dedicated heart failure staff – heart failure nurse, heart failure dietician.’ (Cardiologist-24)

	Lack of counsellor or patient care coordinator	‘The things which are missing are you know there is a concept something called as a counsellor which is not there too much in Indian health infrastructure.’ (Physician-02)

	Lack of robust communication systems	‘Firstly, I think there is lot of misguidances to the patient, as to how, when and where they should approach which specialty. That is the thing that is not fixed in our country.’ (Physician-09)

	Poor referral linkages	‘Unless the patient is very sick and they (private clinics) want to get rid of the patient or if the patient in due course become sick they refer otherwise they keep the patient and after that once the patient spends 60-70 thousand rupees they send the patient for government scheme, there we are supposed to treat the patient, do angioplasty, everything it is very difficult for us so we can’t tell it openly because if you tell it openly that doctor is not going to refer to you at all.’ (Cardiologist-26)

	Lack of monitoring systems to follow-up patients	‘If there is a lot to follow-up we don’t remember also because nothing is computerized.’ (Cardiologist-02)


Further, most physicians recognized that poor patient adherence to medications, polypharmacy and mixed recommendations from other traditional health practitioners influenced chronic care of CVD. For example, a physician stated:

‘The secondary prevention (of CVD) in whom we advise to continue medicines lifelong, but good number of them (patients) tend to stop their medicines three to six months from the time of the index event thinking that they are normal, especially (those patients) on the governmental schemes get procedures done, tend not to continue medicines.’

Affordability of CVD treatment emerged as a greatest challenge from patients’ perspectives. Particularly for those belonging to the lower income groups, including among individuals with low educational attainment, *high cost of treatment* and *low knowledge/awareness* about disease were major barriers. On the other hand, people who are highly educated were thought to be susceptible to unreliable information from online sources according to physicians. The care providers expressed concern that the *misinformation epidemic* circulating on WhatsApp and other social media platforms mislead patients with chronic conditions. For example, one physician stated: ‘There is lot of misinformation campaign goes on WhatsApp … Because of that, lot of mistrust has arisen in patients, and they (patient) just keep on changing doctors, so that misinformation epidemic has to be controlled…that takes lot of time.’ Difficulties in maintaining lifestyle changes, and misconceptions around dietary and exercise habits also emerged as barriers from patient perspectives.

### Barriers and enablers of implementing C-QIP strategy

**Figure 2:**
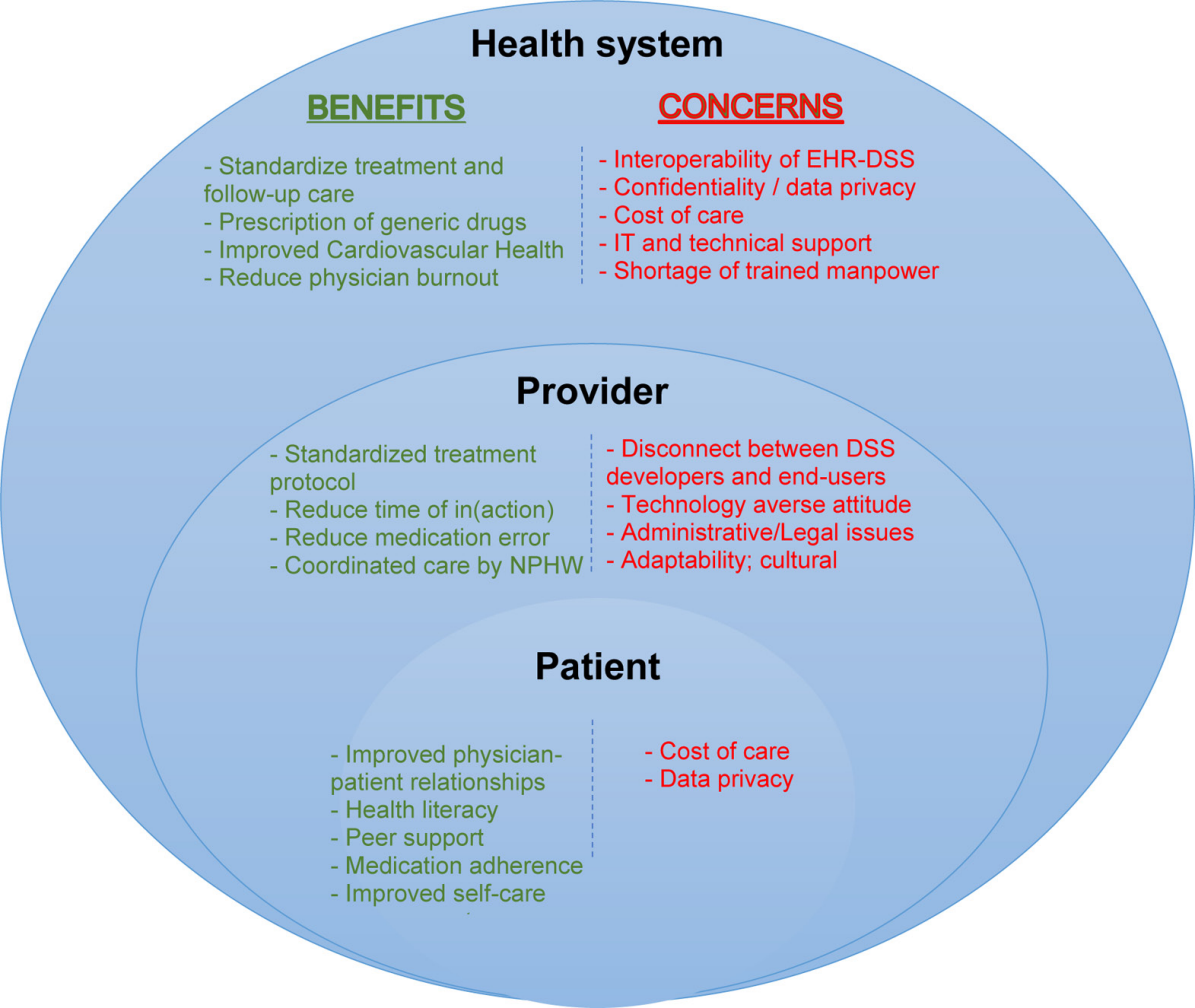
Perceived benefits and concerns related to the C-QIP strategy in India.

**Figure 3:**
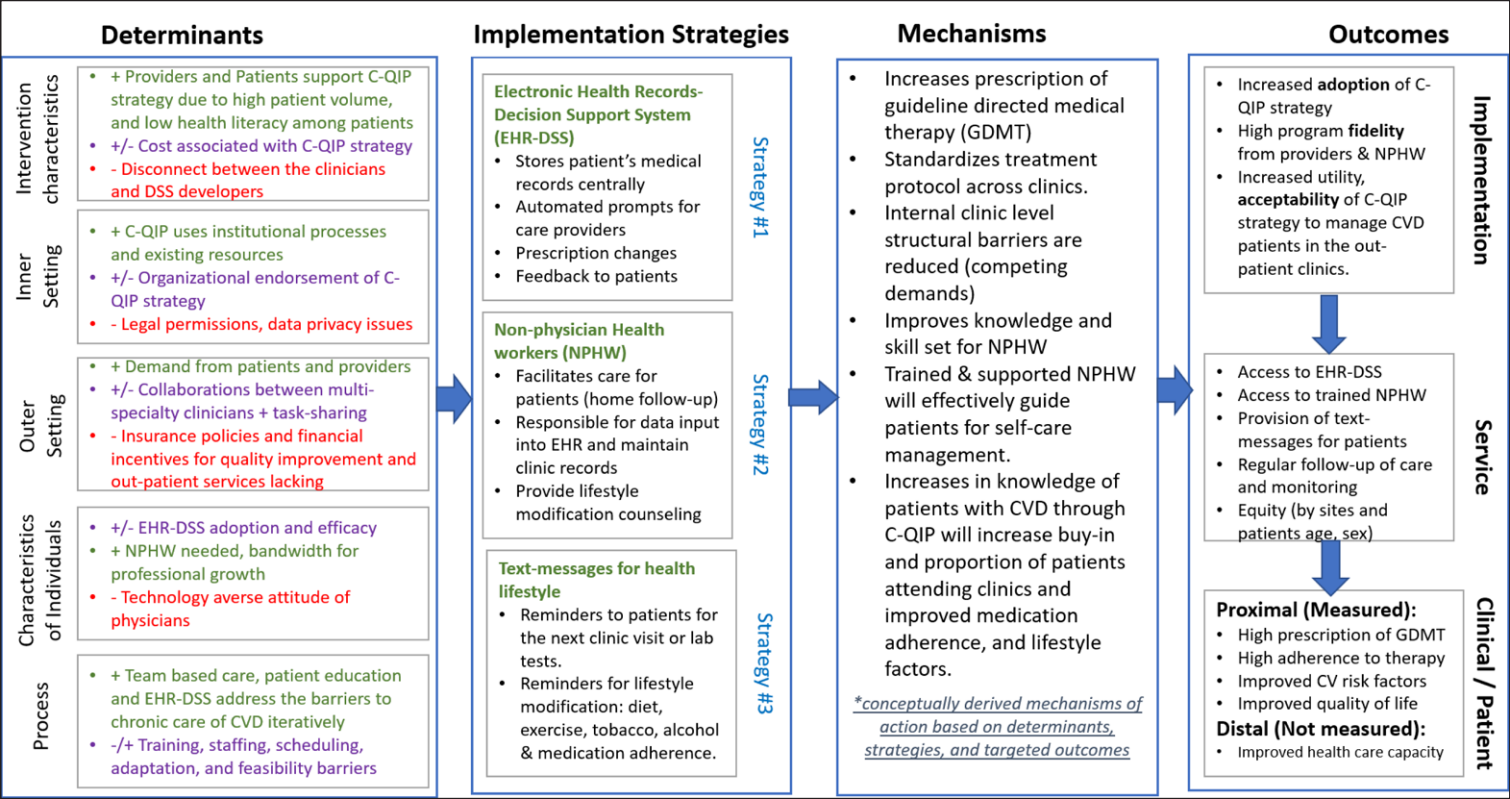
Implementation research logic model for C-QIP strategy. + indicates enablers; - barriers; +/- mixed views (may act as enablers or barriers).

Figure 2 illustrates perceived benefits and concerns around implementing the C-QIP strategy. Further, we mapped the barriers and enablers of implementing the C-QIP strategy using CFIR domains to inform an implementation research logic model depicting the determinants, implementation strategies, hypothesized mechanisms and desired outcomes (Figure 3). The key determinants that influenced the implementation strategies to be evaluated in a prospective feasibility RCT were synthesized using the five domains postulated by CFIR and is discussed further in the following.

### Intervention characteristics

The C-QIP strategy combines clinical decision supported electronic health records, task-sharing via trained NPHW, and patient-facing text messages with healthy lifestyle advice to improve CVD care quality. Physicians thought that implementing the C-QIP strategy would standardize treatment protocols and reduce medication errors as they will receive alerts or clinical decision support prompts for timely treatment modification. This was best summarized by a cardiologist, ‘So, given the busy clinic we (physician) miss out on certain essential prescription drugs which need to be there for heart failure patients for example. This DSS prompt can be an alert for the physician if something is missed in the prescription.’

EHR-DSS may offer additional value as it will endorse prescription of generic drugs, which can reduce out-of-pocket medical expense, and electronically stored patient data can be used for research purpose (e.g., big data analytics) to inform clinical decision making. Further, physicians supported the use of NPHWs to facilitate care for patients and assist in collecting/recording patient data into the EHR, thus allowing more consultation time with patients as described by a physician health administrator, ‘*it (C-QIP) is worthwhile, it is a beautiful activity. I strongly recommend that. 90% of your problems and recurrence will be stopped if you are able to modify the lifestyle, motivate them (patients) to modify their lifestyle*.’

The disconnect between EHR-DSS developers and end users (i.e., health care providers) emerged as a major deterrent to the use of EHR-DSS by the providers. One physician stated: ‘Majority of them [EHR-DSS] are designed by non-medicals and they are designed in the IT lead offices who have really not visited the doctors. Their [software developers] advisors are not the real-time doctors, and they have not sat through in the clinic.’ Further, few physicians had concerns about the fidelity of physicians’ use of the EHR-DSS given the varied CVD manifestations, and prognostic factors. Assistance from NPHWs for patient intake and entry into EHR was deemed critical for the success of C-QIP strategy as stated by a physician: ‘Although some hospitals have introduced all this [EHR-DSS] in the outstation, I believe these physicians just do not use the computer, because to just enter all this data, it takes so much time, I am not going to do it.’

Text message-based reminders were thought to be useful and acceptable to both providers and patients as it may increase patients’ retention in care through follow-up visits as well as improve adherence to prescribed therapy. This was best supported by a nurse: ‘[E]ven a person in village today has a mobile, he [patient] has a WhatsApp, who is capable of reading anything in it.’ And, a patient described that, ‘If I get morning message that you will have to take care of your health, I will feel very good.’ To improve the usability and acceptability of text messages, key informants suggested to make it available in the local language.

### Inner setting

All four study sites selected for the feasibility trial lack an EHR-DSS system at baseline, use NPHWs in a minimal capacity, and do not use text messages to support patients’ self-care and management. Physicians had concerns about obtaining legal permission, patient confidentiality, and data privacy issue with the introduction of EHR-DSS as described by a cardiologist: ‘it is very difficult to manage and convince the “so called” CEO (health administrators) of this system with several stakeholders that is the biggest challenge (to) implementing it.’

However, most key informants described that the hospital leadership strongly supports clinic change efforts to improve quality and will provide necessary support in terms of financial resources, training, staffing, equipment, and materials to cater to patients’ needs and improve quality of care.

### Outer setting

Almost all providers and administrators believed the C-QIP strategy should address patient barriers (e.g., low health literacy, cost of care, and poor adherence to pharmacotherapy) and provide patient choices about various services in response to patient needs as a part of the C-QIP strategy. Stakeholders emphasized that to increase the acceptance of NPHW facilitated care, a cultural shift and sensitization along with policy-level changes related to NPHW scope of practice regulations are needed. Physicians reported understanding the needs of their patients. as expressed by a private clinic physician: ‘typically, even today patients and relatives do expect the medical doctors to do this job (counselling) for them.’ Another private practitioner reflecting on known barriers to patient care stated, ‘There could be certain barriers because the patient and his family may not like it (NPHW delivered care). But maybe, we can make it change (by cultural shift) and that should be the way.’ If the C-QIP strategy is found to be effective, it can complement the existing infrastructure and activities emanating from the national program for prevention and management of CVD in India.

### Characteristics of individuals

Providers had mixed views about the EHR-DSS adoption and effectiveness but strongly believed in the involvement of NPHWs to provide patient counselling/education and text messages for chronic care of CVD. A few senior consultants also opposed the use of EHR-DSS as quoted by a cardiologist, ‘Decision support systems are something for people who don’t have their own algorithms in their own mind. For most of the people who have grey hair, they will actually reject them.’

Technology-averse attitudes of some physicians, slow typing speed, and less familiarity with computer interface were quoted as barriers to the wider acceptability and adoption of EHR-DSS as concerned by a physician: ‘They (physicians) would be so resistant to use computers [EHR-DSS] because it takes time for learning.’ Health administrators expressed that dedicated human resources (i.e., NPHWs) and information technology and administrative support would be necessary to effectively implement the C-QIP strategy.

### Process of implementation

This formative, qualitative study is part of a multi-step exploration and preparation process that included a scoping review, multi-stakeholder interviews, and an expert consultation meeting to inform the development and implementation of the C-QIP strategy. The C-QIP strategy was perceived as a complex intervention because it involves multi-level implementation strategies, implementation actors, and integration of different disciplines. The complexity of the intervention drives the need for a clear plan to engage all implementation actors, educate them about the intervention, and encourage the adoption of the C-QIP strategy, as captured by a cardiologist:

‘(In) tertiary care system where there is a lot of disbelief for newer strategies while some people are very forthcoming to the top technologies, there are some who are absolutely against it because that (technology) will take away the human angle from the (health) care system…’

To reduce the complexity of the C-QIP strategy, key informants suggested to provide standard operating procedures and have uniformity in data collection, entry, and execution of DSS plan. The coupling of policy change and the use of NPHWs as intervention champions was perceived as one way to help create an environment that facilitates implementation of the C-QIP strategy. As quoted by a physician administrator:

‘I sincerely hope that because we have introduced DSS in the National program (for CVD, Diabetes, and Hypertension) so I hope it takes up, but I think there is a lot of push back from physicians to such systems…we need physician assistant to help with that work (manage EHR-DSS) that will help bring these things in a more structured way and address CVD burden more efficiently.’

Physicians, health administrators and patients were willing to change and adopt the C-QIP strategy as they believed it will mitigate several structural and systemic barriers to CVD care, such as lack of patient counsellors, poor referral linkages, lack of monitoring systems, and low health literacy among patients.

## Discussion

This multi-stakeholder qualitative analysis revealed several barriers to CVD management such as the lack of QI strategies and policies to promote retention, continuity of care, and minimize costs of outpatient care. Further, the most salient modifiable barriers to chronic care of CVD were structural (i.e., treatments were often too expensive, people could not implement lifestyle changes due to non-supportive environments, systems or policies) and educational (i.e., people understood little about CVD care and navigated multiple channels of health education), which are consistent with previous studies [25,26]. Physicians described how difficult it was due to the amount of care for many patients and were constrained by time, exhaustion, and focus on treatment as opposed to prevention. Providers’ lack of identification of their potential role in low quality care—and in improving the quality of CVD care—seems related to very high clinical loads and potential burnout.

Previous research illustrates the critical role of planning and organizing to identify and mitigate potential pitfalls that may hinder QI strategies before implementation [27,28]. We found team-based care involving NPHW, EHR-DSS, and text-messages were perceived as high value QI strategy to improve chronic care of CVD in low-resource settings like India. Successful implementation of the C-QIP strategy will require adequate human resources (qualified and trained NPHW to support team-based care, patient education, which cannot be achieved by physicians alone), communication systems (EHR for referral linkages and patient follow-up), and financing (ensuring that patients can engage in the C-QIP strategy at a low financial cost). The role of NPHW in care delivery cannot be over emphasized as shown in the CARRS Translation Trial where the EHR-DSS implementation complexities were overcome by the NPHW who collected and entered patients’ data for the DSS, and provided a printed copy of DSS diabetes management plan for physicians’ review [29].

C-QIP strategy represents an important extension to the existing care models by involving trained NPHWs who can play a larger role than what is currently allowed within their scope of practice. NPHW could obtain recommendations from specialists and convey treatment plans to patients, though the potential for miscommunication exists with additional actors. Whether they provide education to patients for self-care management or more prescriptive activities, NPHWs could serve as the fulcrum of care by ensuring continuity of care for patients through coordination between providers and patients using EHR-DSS and text messages. Patients perceived that C-QIP strategy will empower them for better self-care, improve their knowledge about disease and prevention, and increase their trust in the care providers. However, patients were concerned about the financial viability of such a QI strategy since cost of treatment is a priority (major barrier) than maintaining continuity of care.

In this study, hospital administrators believed that the C-QIP strategy can standardize care delivery and enhance patient self-care management. The support of hospital-level leadership and culture plays a critical role in the implementation of QI strategy as also learned in our previous experience of conducting process evaluation of the ACS QUIK trial, which suggest that implementation and acceptability of a QI toolkit were enhanced by hospital-level management support that leveraged available resources to implement the toolkit [30]. Major implementation challenges identified in this study were interoperability of EHR-DSS, the fear that by introducing an EHR-DSS could take away physicians’ opportunities for teaching junior doctors by review of manually prepared patient case reports, and the disconnect between EHR-DSS developers and treating physicians, which will be overcome by using a co-design strategy involving providers and patients throughout the stages of intervention design ideation, prototype testing, and implementation [31,32,33]. For example, a 2015 study from Kenya demonstrated how researchers engaged in the entire care cascade across all health system levels by utilizing community resources, task-sharing, integrated health record, and clinical decision support to improve access to high-quality, and sustainable care for CVD [34]. The C-QIP strategy, which is yet to be evaluated in a RCT, may have similar effects on multiple targets: patient education and self-care, adherence/prescription of guideline-directed medical therapy, and coordinated teams and systems to follow-up patients.

### Strengths and limitations

Through the analysis and interpretation of in-depth interviews with multiple stakeholders, we have provided insights into the current practices, challenges, and opportunities around CVD care in a low resource setting such as India. Further, our results shed light on how the interaction of different CFIR domains (intervention characteristics, inner setting, outer setting, characteristics of individuals) could guide the successful implementation of C-QIP strategy. In particular, the findings of this study described the barriers and facilitators to implementing the C-QIP strategy. A unique strength of this study is that it does not focus on providers and health administrators’ perspective but also include experiences of patients and care givers.

This study has few limitations. First, we focused on a breadth of views from diverse health system stakeholders across diverse settings as opposed to studying a single clinical setting. Because providers and patients in these setting face different challenges, the diluting of context within or across clinical settings may cause us to overlook certain facilitators or barriers to a program that might be seen when focusing explicitly on a smaller number of clinical settings. Second, patients, caregivers, and health administrators were selected from the four participating sites in the C-QIP feasibility trial, which might introduce the selection and social desirability biases. However, we utilized a purposive sampling frame to recruit key informants to achieve diverse representation and glean different perspectives on enablers and facilitators of the C-QIP strategy in India. Third, our findings are based on in-depth interviews with little observation using ethnographic methods, which could have provided insights into the actual clinic flow, dynamic interactions between clinicians, nurses, pharmacists, and patients, and process of care measures, so this study is largely based on the self-reported participants’ views and experiences.

## Conclusions

Our study identified enablers and barriers to implement C-QIP strategy in low resource settings in India. The modifiable barriers were low health literacy of patients, high patient volume, too few specialists, physician burnout and time-constraints, and lack of robust communication, and referral systems. Team-based care involving NPHW, patient education, and EHR-DSS emerged as potentially useful strategies to improve quality of care among patients with CVD. C-QIP strategy has the potential to improve process of care measures, clinical outcomes, and patient experiential quality.

## Data Availability Statement

Corresponding author has access to all study data. Data will be made available to the external researchers upon request.

## Additional File

The additional file for this article can be found as follows:

10.5334/gh.1161.s1 Supplementary file. COREQ (COnsolidated criteria for REporting Qualitative research) Checklist.
